# Thyrotropic Axis and Disorders of Consciousness in Acquired Brain Injury: A Potential Intriguing Association?

**DOI:** 10.3389/fendo.2022.887701

**Published:** 2022-07-07

**Authors:** Chiara Mele, Antonio De Tanti, Sergio Bagnato, Lucia Francesca Lucca, Donatella Saviola, Anna Estraneo, Pasquale Moretta, Laura Marcuccio, Bernardo Lanzillo, Gianluca Aimaretti, Antonio Nardone, Paolo Marzullo, Valeria Pingue

**Affiliations:** ^1^ Department of Clinical-Surgical, Diagnostic and Pediatric Sciences, University of Pavia, Pavia, Italy; ^2^ Cardinal Ferrari Centre, Santo Stefano Riabilitazione KOS-CARE, Fontanellato, Parma, Italy; ^3^ Unit of Neurophysiology and Unit for Severe Acquired Brain Injuries, Rehabilitation Department, Giuseppe Giglio Foundation, Cefalù, Italy; ^4^ Research in Advanced Neurorehabilitation (RAN), S. Anna Institute, Crotone, Italy; ^5^ Department of Neurorehabilitation for Severe Acquired Brain Injury, Don Carlo Gnocchi Foundation, Scientific Institute for Research and Health Care, Florence, Italy; ^6^ Neurology Unit, Santa Maria della Pietà General Hospital, Nola, Italy; ^7^ Istituti Clinici Scientifici Maugeri IRCCS, Neurorehabilitation Unit of Telese Terme Institute, Telese Terme, Italy; ^8^ Department of Translational Medicine, University of Piemonte Orientale, Novara, Italy; ^9^ Istituti Clinici Scientifici Maugeri IRCCS, Neurorehabilitation and Spinal Unit of Pavia Institute and Neurorehabilitation Unit of Montescano Institute, Pavia, Italy; ^10^ Istituto Auxologico Italiano, IRCCS, Laboratory of Metabolic Research, S. Giuseppe Hospital, Piancavallo, Italy

**Keywords:** thyrotropic axis, disorders of consciousness, neurorehabilitation, functional outcome, recovery

## Abstract

**Purpose:**

A potential involvement of thyrotropic axis in influencing the state of consciousness could be hypothesized. We aimed at investigating thyroid function tests as predictors of disorders of consciousness (DoC) and relating recovery in a large cohort of patients with DoC secondary to acquired brain injury (ABI).

**Methods:**

This retrospective, multicenter, cohort study included 151 patients with DoC following ABI, consecutively admitted for a 6-month neurorehabilitation program. Data on etiology of brain injury, evolution of DoC, disability and rehabilitation assessments, and death during rehabilitation were collected at baseline and on discharge. Thyroid function tests (serum TSH, fT4 and fT3 levels) were assessed on admission in all patients and at final discharge in 50 patients.

**Results:**

Lower baseline TSH levels and greater TSH increments (ΔTSH) after neurorehabilitation predicted a favorable change in DoC independent of age, sex, BMI, etiology of brain injury and initial DoC subtype (TSH: OR=0.712, CI 95% 0.533-0.951, p=0.01; ΔTSH: OR=2.878, CI 95% 1.147-7.223, p=0.02). On the other hand, neither fT4 nor fT3 or their variations appeared to play any role on DoC changes after 6-months inpatient neurorehabilitation. A lower magnitude of ΔfT4 acted as a strong predictor of improved functional disability level (β=0.655, p=0.002) and cognitive functions (β=-0.671, p=0.003), implying that smaller changes in fT4 were associated with higher outcomes.

**Conclusions:**

Serum TSH levels assessed in the subacute post-ABI phase and its variation during neurorehabilitation could represent a potential biomarker of DoC evolution, while variations in fT4 levels seem to be associated with rehabilitation and cognitive functions. Further studies are needed to investigate the mechanisms underlying these associations.

## 1 Introduction

Consciousness is regulated by a complex series of mechanisms involving several neural structures (1–3). The ascending reticular activating system (ARAS) has been considered as the main neural structure for consciousness. The ARAS is a complex network that links a portion of brainstem reticular formation with thalamic nuclei, the basal forebrain, hypothalamus, and the cerebral cortex (1, 2). Prolonged disorders of consciousness (DoC) represent states of altered consciousness and are categorized into two main conditions: 1) vegetative state (VS), defined as an unconscious, dissociative state of wakefulness without awareness, and 2) minimally conscious state (MCS), characterized by evidence of wakefulness and partial preservation of awareness (4, 5). The presence of DoC in patients with severe brain injury, including both traumatic and non-traumatic etiology, suggests a transient dysfunction or injury of ARAS (6, 7), with the withdrawal of excitatory synaptic activity of the cerebrum produced by deafferentation or disfacilitation of neocortical, thalamic and striatal neurons (8–10).

Despite the growing evidence about the potential role of neuroinflammation, seizures and metabolic alterations in influencing the state of consciousness (11–13), the pathogenetic mechanisms underlying DoC have not been fully elucidated yet. Moreover, besides the robust predictive value of somatosensory evoked potentials in anoxic patients (14), no additional reliable predictive biomarker of recovery of consciousness has been identified.

A potential involvement of thyroid hormones (THs) in influencing the state of consciousness could be hypothesized. THs are essential for the functions of central nervous system (CNS). Such role in the brain homeostasis emerges from the neurological complications of hypothyroidism in both infants and adults (15). Some case reports described consciousness impairments resulted from severe hypothyroidism or hyperthyroidism with thyroid storm (16–18). It is known that THs signaling influences activation of ARAS and that thyrotropin releasing hormone (TRH) is able to increase ordinary consciousness during the day and nonordinary consciousness in the night (19).

To date, there are no data about the potential association between thyroid function and recovery in euthyroid patients with prolonged DoC. Therefore, this study was designed to investigate the role of thyroid function as a potential predictive biomarker of recovery in a large cohort of patients with prolonged DoC secondary to acquired brain injury (ABI).

## 2 Methods

### 2.1 Study Design and Population

This observational, retrospective, multicenter, cohort study included 151 patients admitted to five Italian centers specialized in the rehabilitation of patients with DoC following acute brain injury between June 2019 and March 2021. Coordinating center was Istituti Clinici Scientifici (ICS) Maugeri, institute of Pavia.

Eligibility criteria included: 1) age ≥ 18 years; 2) clinical diagnosis of prolonged DoC secondary to ABI; 3) post-injury time ranging from 28 days to 3 months; 4) admission to a rehabilitation unit for a 6-month neurorehabilitation program. Exclusion criteria were the presence of pre-existing brain injury occurred before the index date, previously known hypothalamic–pituitary dysfunctions or neurological diseases, known thyroid disease, use of levothyroxine (L-T4) or triiodothyronine (T3) and/or use of medications potentially interfering with thyroid function.

Following the acute event and post-acute hospitalization according to routine management, all patients diagnosed with DoC included in our dataset underwent a 6-month inpatient neurorehabilitation program consisting of individual 3-h daily treatment cycles performed 6 days per week that included nursing assistance, physiotherapy, occupational therapy, speech therapy, cognitive training, nutrition assistance, as well as neuropsychological and social support.

The study design was conformed to the ethical guidelines of the Declaration of Helsinki and was approved by the local Ethical Committee ICS Maugeri (#2503 CE). The participants’ authorized representatives signed a written informed consent.

### 2.2 Variables, Data Sources and Measurements

At the time of the study inclusion according to the previously mentioned study criteria, all participating centers collected the following demographic and clinical informations: sex, age, etiology of brain injury, time after injury, medical comorbidities before the brain injury, as assessed by the Cumulative Illness Rating Scale (CIRS) and expressed by a severity index (mean of scores for 13 body system items, each score 1 to 5 depending on severity; range 13-65) and a comorbidity index (number of scores ≥3).

Both on admission (T0) and at discharge (T1), the following variables were evaluated: thyroid function, body mass index (BMI), and outcome measure including Coma Recovery Scale-Revised (CRS-R) (20), Glasgow Outcome Scale-Extended (GOS-E) (21), Disability Rating Scale (DRS) (22), and Functional Independence Measure (FIM) scale (23, 24).

#### 2.2.1 Thyroid Function Test

Routine analysis and thyroid function tests (TSH, fT4, fT3) were assessed on admission for rehabilitation for all patients. In 50 patients, thyroid functions tests were repeated upon discharge from the 6-month rehabilitation program. Serum samples were assayed for fT4, fT3 and TSH using an automated chemiluminescence assay system (Immulite 2000; DPC, Los Angeles, CA). The principle of the method is a two-site, solid-phase chemiluminescent immunometric assay (TSH) or competitive immunoassay (fT4 and fT3). The reference ranges are 0.4–4.0 µIU/mL for TSH, 0.8–1.8 ng/dL for fT4, and 1.8–4.2 pg/mL for fT3.

#### 2.2.2 Body Measurements

Weight and height were measured to the nearest 0.1 kg and 0.1 cm, respectively. BMI was expressed as body mass (kilograms)/height (meters)^2^.

#### 2.2.3 Outcome Measures

Within one week since beginning of rehabilitation and study entry, all patients underwent standard neurological examination and assessment with the Italian version of the CRS-R over three consecutive days, with the CRS-R assessed at least five times. The CRS-R is used to diagnose VS, MCS, and emergence from MCS and is the most reliable tool available for the assessment of consciousness in patients with DoC following coma (20, 25, 26). It consists of 23 hierarchically organized items grouped into six subscales addressing auditory, visual, motor, oromotor/verbal, communication, and arousal functions. Total CRS-R scores range from 0 (comatose state) to 23 (emergence from MCS) (26, 27). The same 3-day evaluation with five CRS-R administrations was repeated 6 months after brain injury.

Six-month outcomes were characterized using the GOS-E, a global 1–8 scale with the categories of death (score of 1), vegetative state, severe disability (lower and upper), moderate disability (lower and upper), and good recovery (lower and upper; scores of 7 and 8) (21).

Level of functional disability was evaluated on admission and at discharge using DRS total score, an eight-items scale which addresses all three World Health Organization (WHO) categories: impairment, disability, and handicap (22).

Rehabilitation outcomes were evaluated through the FIM scale, an 18-item measurement tool that explores individual’s physical, psychological and social function (23, 24). The tool is used to assess the patient’s level of disability as well as change in patient status in response to rehabilitation or medical intervention (28).

### 2.3 Statistical Analysis

Values are expressed as median and interquartile range (IQR), or absolute number and percentage at the beginning and at the end of the study, and calculated as absolute variations over baseline values (Δ=T1-T0). Data were tested for normality of distribution by the Shapiro–Wilk test and log-transformed when needed, to correct for skewness. For comparative analysis, Mann–Whitney U-test between groups and Wilcoxon test within groups were used. Spearman’s correlation analysis and χ2 were used to identify significant associations between variables of interest. Multivariate logistic regression analysis was used to identify the independent predictors of DoC evolution after a 6-months inpatient neurorehabilitation program. Odds ratio (OR), 95% confidence interval (95% CI) and related significant values obtained from regression are reported. Univariate linear regression analysis was conducted to test the potential predictive role of thyroid function parameters on recovery and functional outcome, whereas multivariate linear regression models were built to evaluate the independent predictors of variation in recovery and functional scales in the population as a whole. The multilinear models included combinations of independent variables encompassing age, sex, BMI, diagnosis of DoC on admission, etiology of brain injury and thyroid function parameters. β coefficients and related significance values obtained from the models are reported. P < 0.05 was considered as statistically significant. Statistical analyses were performed using SPSS version 21 (Somers, NY, USA).

## 3 Results

### 3.1 Clinical Characteristics and Outcomes

A summary of clinical and functional characteristics of the study population is reported in **Table 1**. The male-to-female ratio was 2:1 and the median age at diagnosis was 58.9 (IQR, 48.1-70.9) years. Median CIRS score was 0.31 (IQR, 0.15-0.62). On admission to rehabilitation, VS was diagnosed in 94 patients (62.3%) and MCS in 57 cases (37.7%). DoCs were mainly caused by haemorragic stroke (37.1%) and traumatic brain injury (TBI) (29.8%). After 6-month inpatient neurorehabilitation program, an improvement of the DoC was documented in 53 patients (35.1%). In particular, a recovery of full consciousness was observed in 31 patients (20.5%), and transition from a VS to MCS in 22 cases (23.4%). Worsening of the DoC was observed in only 5 patients (4.1%). Death occurred in 25 patients (16.6%) within 6 months of brain injury.

**Table 1:** Clinical and functional characteristics of the study population on admission (T0) and at discharge (T1) as a whole and after subgrouping according to the diagnosis of DoC on admission (MCS vs VS) and the change in DoC after 6-months neurorehabilitation program (improved DoC vs unchanged DoC).

Variables		Whole population(n=151)	DoC T0		DoC T1	
			MCS (n=57)	VS (n=94)	Improved DOC (n=53)	Unchanged DOC (n=73)
		N (%) ormedian (IQR)	N (%) ormedian (IQR)	N (%) ormedian (IQR)	N (%) ormedian (IQR)	N (%) ormedian (IQR)
Sex	Male	99 (65.6)	38 (66.7)	61 (64.9)	37 (69.8)	46 (63.0)
	Female	52 (34.4)	19 (33.3)	33 (35.1)	16 (30.2)	27 (37.0)
Age (years)		58.9 (48.1-70.9)	58.5 (48.0-72.0)	59.4 (48.2-70.7)	56.7 (39.0-68.7)	59.5 (50.3-71.1)
BMI	T0	**23.1 (20.4-25.0)**	22.7 (20.5-24.8)	23.4 (20.2-25.6)	22.2 (20.8-24.8)	23.1 (20.1-25.7)
	T1	**22.8 (20.4-26.0)*^§§^***	22.7 (21.1-25.9)	22.9 (19.8-25.9)	23.2 (21.0-25.3)	22.5 (19.9-26.3)
	Δ	-0.04 (-4.4-0.4)	0.0 (-1.9-1.2)	-0.4 (-6.1-0.2)	0.0 (-0.6-1.9)	0.0 (-1.9-0.3)
Etiology	Anoxic	33 (21.8)	**5 (8.8)**	28 (29.8)**	**6 (11.3)**	23 (31.5)**
	Haemorrhagic	56 (37.1)	20 (35.1)	36 (38.3)	18 (34.0)	28 (38.4)
	Ischaemic	17 (11.3)	9 (15.8)	8 (8.5)	7 (13.2)	6 (8.2)
	TBI	45 (29.8)	**23 (40.3)**	22 (23.4)*	**22 (41.5)**	16 (21.9)*
Diagnosis T0	VS	94 (62.3)	**-**	**-**	32 (60.4)	46 (63.0)
	MCS	57 (37.7)	**-**	**-**	21 (39.6)	27 (37.0)
Diagnosis T1	VS	51 (33.8)	**5 (8.8)**	46 (48.9)***	**0 (0.0)**	51 (69.9)***
	MCS	44 (29.1)	**22 (38.6)**	22 (23.4)*	22 (41.5)	22 (30.1)
	SC	31 (20.5)	**21 (36.8)**	10 (10.7)***	**31 (58.5)**	0 (0.0)***
	Death	25 (16.6)	9 (15.8)	16 (17.0)	–	–
CRS-R	T0	**6.0 (4.0-9.0)**	**10.0 (9.0-11.5)**	4.0 (2.0-6.0)***	**6.0 (4.0-9.3)**	5 (3.0-9.0)
	T1	**9.0 (5.0-18.0)*^§§§^***	**14.5 (10-22.3)*^§§§^***	6.0 (4.0-11.0)***,*^§§§^*	**19.0 (11.3-23)** *^§§§^*	6.0 (4.0-9.0)***
	Δ	2.0 (0.0-7.3)	2.5 (0.0-8.0)	2.0 (0.0-7.0)	**9.0 (5.3-14.0)**	1.0 (0.0-2.0)***
CIRS Total score		0.31 (0.15-0.62)	0.27 (0.08-0.52)	0.38 (0.15-0.69)	0.38 (0.08-0.77)	0.31 (0.15-0.54)
GOS-E	T0	**2 (2-2)**	**2 (2-3)**	2 (2-2)***	**2 (2-3)**	2 (2-2)
	T1	**2 (2-3)*^§§§^***	**3 (2-3)*^§^***	2 (2-3)***,*^§^*	**3 (3-4)** *^§§§^*	2 (2-2)***
	Δ	0 (0-1)	0 (0-1)	0 (0-0)	**1 (0-1)**	0 (0-0)***
DRS	T0	**24.0 (23.0-26.0)**	**22.5 (21.8-24.0)**	26.0 (24.0-26.0)***	**24.0 (23.0-26.0)**	25.0 (23.8-26.0)*
	T1	**22.5 (18.0-25.0)*^§§§^***	**21.0 (13.5-22.0)*^§§§^***	24.0 (20.0-26.0)***,*^§§§^*	**16.0 (12.5-21.0)** *^§§§^*	24.0 (22.0-26.0)***^,§^
	Δ	-2.0 (-6.8-0.0)	-2.0 (-7.0-0.0)	-2.0 (-5.0-0.0)	**-7.0 (-10.0–3.09)**	0.0 (-2.0-0.0)***
FIM	Tot T0	**18 (18-18)**	**18 (18-20)**	18 (18-18)***	**18 (18-18)**	18 (18-18)
	Tot T1	**20 (18-36)*^§^***	**28 (19-68)*^§§§^***	18 (18-22)***,*^§^*	**33 (21-69)** *^§§§^*	18 (18-19)***
	Δ Tot	1 (0-17)	**10 (0-45)**	0 (0-4)*	**13 (3-49)**	0 (0-0)***
	Mot T0	13 (13-13)	13 (13-13)	13 (13-13)	**13 (13-13)**	13 (13-13)
	Mot T1	13 (13-20)	**17 (13-47)*^§§§^***	13 (13-13)**,*^§^*	**20 (13-49)** *^§§§^*	13 (13-13)***
	Δ Mot	0 (0-7)	**4 (0-33)**	0 (0-0)**	**6 (0-34)**	0 (0-0)***
	Cog T0	**5 (5-5)**	**5 (5-7)**	5 (5-5)***	**5 (5-5)**	5 (5-5)
	Cog T1	**7 (5-12) *^§§§^***	**10 (6-19)*^§§§^***	5 (5-9)**,*^§§^*	**11 (8-24)** *^§§§^*	5 (5-6)***
	Δ Cog	1 (0-7)	**4 (0-14)**	0 (0-4)*	**6 (3-16)**	0 (0-0)***

Data are expressed as median and interquartile range (IQR) or absolute number and percentage.

Comparison between groups were performed with χ2 or Mann–Whitney U tests, *p<0.05, **p<0.01, ***p<0.0001.

Comparison between T0 and T1 was performed by Wilcoxon test, ^§^p<0.01, ^§§^p<0.001, ^§§§^p<0.0001. Significant differences are shown in bold characters.

DoC, disorder of consciousness; T0, on admission; T1, at discharge; BMI, body mass index; VS, vegetative state; MCS, minimally conscious state; SC, state of consciousness; TBI, traumatic brain injury; CRS-R, Coma Recovery Scale-Revised; CIRS, Cumulative Illness Rating Scale; GOS-E, Glasgow Outcome Scale-Extended; DRS, Disability Rating Scale; FIM, Functional Independence Measure.

When the population was subgrouped according to the diagnosis of DoC on admission and after 6-month neurorehabilitation, there were no differences in sex, age, BMI and comorbidities (**Table 1**). In terms of brain injury etiology, patients with VS on admission and those with unchanged DoC at discharge showed a higher prevalence of anoxic lesions, while those with MCS on admission as well as those with improved DoC at discharge had suffered more frequently from TBI as compared to their respective counterparts.

As expected, patients with VS on admission and those with unchanged DoC at discharge had a worse disability level, i.e. DRS, as well as functional, i.e. FIM, and global outcomes, i.e. GOS-E, than subjects with MCS on admission or those with improved DoC at discharge, respectively.

After neurorehabilitation, significantly decreased DRS, improved FIM and GOS-E scores were documented both in the whole population and in subgroups, except for patients with unchanged DoC at discharge (**Table 1**).

### 3.2 Thyroid Function and DoC

The results of thyroid function testing on admission (T0) and at discharge (T1) are summarized in **Table 2**. At baseline, TSH levels were below the reference range in 9.3% of patients and were normal in the remaining 90.7% of cases. All patients with low TSH levels showed normal fT4 levels, while low fT3 levels were seen in one case. In patients with normal TSH, two cases had low fT4 levels and 26 subjects (18.9%) had low fT3 levels. No association was found between TSH or fT4 levels with age, sex, BMI and etiology of brain injury. In contrast, fT3 levels decreased with increasing age (rho=-0.20, p=0.02). An association related fT4 and fT3 (rho=0.29, p=0.001), while none was correlated with TSH levels.

**Table 2:** Thyroid function testing in the population as a whole and subgrouped according to DoC diagnosis on admission and at discharge.

Variables		Whole population (n=151)	DoC T0		DoC T1	
			VS (n=94)	MCS (n=57)	Improved DoC (n=53)	Unchanged DoC (n=73)
		Median (IQR)	Median (IQR)	Median (IQR)	Median (IQR)	Median (IQR)
TSH (µIU/mL)	T0	1.59 (0.91-2.63)	1.55 (0.79-2.74)	1.75 (1.28-2.51)	**1.46 (1.04-2.07)**	1.94 (0.85-3.27)*
	T1^&^	2.11 (1.29-3.05)	2.13 (1.05-3.52)	2.01 (1.63-2.47)	1.91 (1.29-2.40)	2.44 (1.33-4.60)
	Δ^&^	0.00 (-0.93-0.94)	0.00 (-0.97-0.91)	0.24 (-0.34-0.94)	**0.24 (-0.26-1.07)**	-0.26 (-1.17-0.80)**
fT4 (ng/dL)	T0	1.29 (1.10-1.52)	1.31 (1.10-1.53)	1.29 (1.14-1.51)	**1.19 (1.09-1.50)**	1.35 (1.11-1.51)
	T1^&^	1.17 (0.96-1.39)	1.22 (0.96-1.43)	1.10 (0.96-1.27)	**1.04 (0.93-1.23)*^§§^***	1.26 (1.13-1.44)
	Δ^&^	-0.01 (-0.17-0.15)	-0.02 (-0.17-0.12)	0.01 (-0.15-0.21)	-0.12 (-0.24-0.003)	0.12 (-0.01-0.25)
fT3 (pg/mL)	T0	**2.25 (1.87-2.72)**	2.22 (1.82-2.74)	2.31 (1.88-2.64)	2.27 (2.03-2.73)	2.32 (1.74-2.65)
	T1^&^	**2.55 (2.08-2.96)*^§^***	2.41 (1.91-2.92)	2.77 (2.55-3.01)	2.90 (2.27-3.01)	2.41 (1.94-2.60)
	Δ^&^	0.26 (0.00-0.92)	0.12 (0.00-0.67)	0.60 (0.05-1.12)	0.43 (0.00-0.98)	0.17 (0.01-0.72)

Data are expressed as median and interquartile range (IQR).

^&^Data available for 50 patients.

Comparison between groups were performed with Mann–Whitney U-tests, *p<0.05, **p<0.01.

Comparison between T0 and T1 was performed by Wilcoxon test, ^§^p<0.05, ^§§^p<0.01.

Significant differences are shown in bold characters.

DoC, disorder of consciousness; T0, on admission; T1, at discharge; VS, vegetative state; MCS, minimally conscious state; TSH, thyroid-stimulating hormone; fT4, free thyroxin; fT3, free triiodothyronine.

Following the 6-month rehabilitation program, thyroid function parameters were available in 50 patients. At this stage, only 3 patients (6.0%) had low TSH levels, with low fT3 in one case. In patients with normal TSH, low fT3 levels were observed in 4 cases (8.0%). All subjects had normal fT4 levels. Thyroid parameters at discharge were not correlated with age, sex, BMI and etiology of brain injury and to each other (data not shown).

When the population was divided by diagnosis of DoC on admission, we found no differences in thyroid function parameters. However, patients with improved DoC exhibited lower starting TSH levels and achieved a greater TSH variation (ΔTSH) after rehabilitation than patients with unchanged DoC (p<0.01). Also, a significant increase in fT3 levels was documented in the whole population (p<0.05), whereas fT4 significantly decreased in the subgroup of patients with an improved DoC at discharge as compared to those with unchanged DoC (p<0.01).

Multivariate logistic regression models were built to identify independent predictors of changes in DoC after neurorehabilitation in the whole population (model 1) and in the subgroups receiving thyroid hormone assessment before and after rehabilitation (model 2). Independently from age, sex, BMI, etiology of brain injury and initial diagnosis of DoC, we found that lower TSH levels on admission and greater ΔTSH were independent predictors of improved DoC (**Table 3**). Neither fT4 nor fT3 or their variations appeared to play any role on DoC changes after 6-months inpatient neurorehabilitation (data not shown).

**Table 3:** Multivariable logistic regression analysis showing independent predictors for DoC evolution in the population as a whole.

Covariates	DoC T1 (Unchanged DoC=0, Improved DoC=1)		
	OR	CI 95%	p-value
**MODEL 1**			
**TSH T0 (µIU/mL)**	**0.712**	**0.533-0.951**	**0.01**
Age (years)	0.982	0.957-1.009	0.20
Sex (M=0, F=1)	0.929	0.376-2.294	0.87
BMI T0 (kg/m^2^)	0.983	0.892-1.085	0.28
Diagnosis T0 (VS=0, MCS=1)	0.960	0.411-2.244	0.92
Etiology (No TBI=0, TBI=1)	1.727	0.642-4.648	0.28
**MODEL 2**			
**ΔTSH (µIU/mL)**	**2.878**	**1.147-7.223**	**0.02**
Age (years)	0.936	0.872-1.004	0.07
Sex (M=0, F=1)	0.819	0.101-6.656	0.85
BMI T0 (kg/m^2^)	1.015	0.786-1.311	0.91
Diagnosis T0 (VS=0, MCS=1)	1.854	0.200-17.199	0.59
Etiology (No TBI=0, TBI=1)	0.580	0.054-6.259	0.65

Significant associations are shown in bold characters.

DoC, disorder of consciousness; T0, on admission; T1, at discharge; TSH, thyroid-stimulating hormone; BMI, body mass index; VS, vegetative state; MCS, minimally conscious state; TBI, traumatic brain injury.

### 3.3 Thyroid Function and Rehabilitation Outcomes

Univariate linear regression analysis was conducted to evaluate the potential predictive role of thyroid function parameters on recovery and functional outcomes. TSH, fT4 and fT3 levels, as well as their variations, were not associated with the recovery and functional outcome in terms of GOS-E, DRS and FIM motor, cognitive and total score (**Supplementary Table 1**).

Multivariate linear regression models evaluated the independent predictors of functional improvements in the population as a whole and in the subgroup of patients having thyroid function tested at discharge. The models achieving the highest coefficient of determination (R2) for each scale are reported in **Tables 4**–**6**. A lower magnitude of ΔfT4 acted as a strong predictor of improved functional disability level (ΔDRS) and FIM cognitive score (ΔFIM), implying that smaller changes in FT4 were associated with higher outcomes. Milder predictors of disability and FIM included DoC on admission and etiology of brain injury (**Tables 4**, **5**).

**Table 4:** Multivariable linear regression analysis showing independent predictors for ΔDRS.

ModelIndependent variables	ΔDRS (R^2^= 0.538)	
	Beta	p-value
Age (years)	-0.125	0.516
Sex (M=0, F=1)	0.037	0.838
BMI T0 (kg/m^2^)	0.148	0.460
**Diagnosis T0 (VS=0, MCS=1)**	**-0.472**	**0.014**
Etiology (No TBI=0, TBI=1)	-0.188	0.343
**ΔfT4 (ng/dL)**	**0.655**	**0.002**

Significant associations are shown in bold characters.

T0, on admission; DRS, Disability Rating Scale; BMI, body mass index; VS, vegetative state; MCS, minimally conscious state; TBI, traumatic brain injury; fT4, free thyroxine.

**Table 5:** Multivariable linear regression analysis showing independent predictors for ΔFIM total, motor and cognitive score.

ModelIndependent variables	ΔFIM total score (R^2^= 0.650)		ΔFIM motor score (R^2^= 0.619)		ΔFIM cognitive score (R^2^= 0.686)	
	Beta	p-value	Beta	p-value	Beta	p-value
Age (years)	0.061	0.757	0.073	0.723	0.007	0.968
Sex (M=0, F=1)	-0.123	0.605	-0.105	0.671	-0.169	0.457
BMI T0 (kg/m^2^)	-0.078	0.721	-0.104	0.651	0.027	0.897
**Diagnosis T0 (VS=0, MCS=1)**	**0.688**	**0.002**	**0.680**	**0.003**	**0.611**	**0.003**
Etiology (No TBI=0, TBI=1)	0.327	0.130	0.352	0.119	0.182	0.357
**ΔfT4 (ng/dL)**	**-0.486**	**0.025**	-0.415	0.059	**-0.671**	**0.003**

Significant associations are shown in bold characters.

T0, on admission; FMI, Functional Independence Measure; BMI, body mass index; VS, vegetative state; MCS, minimally conscious state; TBI, traumatic brain injury; fT4, free thyroxine.

**Table 6:** Multivariable linear regression analysis showing independent predictors for ΔGOS-E.

ModelIndependent variables	ΔGOS-E (R^2^= 0.657)	
	Beta	p-value
Age (years)	-0.489	0.125
Sex (M=0, F=1)	-0.303	0.210
BMI T0 (kg/m^2^)	-0.225	0.331
**Diagnosis T0 (VS=0, MCS=1)**	**0.534**	**0.044**
**Etiology (No TBI=0, TBI=1)**	**-0.709**	**0.046**
ΔTSH (µIU/mL)	-0.233	0.309

Significant associations are shown in bold characters.

T0, on admission; GOS-E, Glasgow Outcome Scale-Extended; BMI, body mass index; VS, vegetative state; MCS, minimally conscious state; TBI, traumatic brain injury; TSH, thyroid-stimulating hormone.

### 3.4 Thyroid Function and Mortality

There were no differences in the prevalence of mortality within the study period between patients with a diagnosis on admission of VS and MCS (**Table 1**). To avoid potential collinearity bias between thyroid function parameters, each parameter entered an individual regression logistic model to assess mortality predictors. Across the three multivariable logistic regression models, age at diagnosis of brain injury (**Table 7**) was identified as the main risk factor for mortality at six months since starting rehabilitation. Baseline TSH, fT4 and fT3 levels were unable to predict mortality in this cohort.

**Table 7:** Multivariable logistic regression models showing the potential risk factors for mortality within 6 months of brain injury in the population as a whole.

Covariates	Death within 6 months of brain injury (No=0, Yes=1)		
	OR	CI 95%	p-value
**MODEL 1**			
TSH T0 (µIU/mL)	1.091	0.810-1.468	0.567
**Age (years)**	**1.055**	**1.019-1.091**	**0.002**
Sex (M=0, F=1)	1.183	0.437-3.199	0.741
BMI T0 (kg/m^2^)	0.981	0.875-1.099	0.738
Diagnosis T0 (VS=0, MCS=1)	0.457	0.251-1.860	0.457
Etiology (No TBI=0, TBI=1)	1.582	0.505-4.950	0.431
**MODEL 2**			
fT4 T0 (ng/dL)	3.114	0.701-13.839	0.136
**Age (years)**	**1.051**	**1.014-1.089**	**0.006**
Sex (M=0, F=1)	1.431	0.518-3.953	0.490
BMI T0 (kg/m^2^)	1.017	0.910-1.137	0.768
Diagnosis T0 (VS=0, MCS=1)	0.526	0.183-1.516	0.235
Etiology (No TBI=0, TBI=1)	2.354	0.678-8.177	0.178
**MODEL 3**			
fT3 T0 (pg/mL)	1.619	0.784-3.347	0.193
**Age (years)**	**1.057**	**1.020-1.096**	**0.003**
Sex (M=0, F=1)	1.176	0.430-3.219	0.752
BMI T0 (kg/m^2^)	1.013	0.906-1.132	0.822
Diagnosis T0 (VS=0, MCS=1)	0.575	0.202-1.631	0.298
Etiology (No TBI=0, TBI=1)	1.854	0.560-6.138	0.312

Significant associations are shown in bold characters.

T0, on admission; TSH, thyroid-stimulating hormone; fT4, free thyroxin; fT3, free triiodothyronine BMI, body mass index; VS, vegetative state; MCS, minimally conscious state; TBI, traumatic brain injury.

## 4 Discussion

The present study investigated the association of thyrotropic axis with DoC and relating recovery in a large cohort of patients with ABI. Our results showed that lower baseline TSH levels and greater TSH increments after neurorehabilitation appears to predict an improvement in DoC independent of age, sex, BMI, etiology of brain injury and initial DoC subtype. On the other hand, smaller variations in fT4 levels appeared to be associated with a greater improvement in functional disability level and cognitive functions.

DoC arise from direct perturbations of neural systems that regulate arousal and awareness, and indirectly from disruptions in the connections between these systems (29). It is known that consciousness is regulated by complex networks involving several neural structures, among which ARAS is considered the most important, as it connects a portion of the brainstem reticular formation with nonspecific thalamic nuclei, the basal forebrain, hypothalamus, and the cerebral cortex (3, 4). All severe ABIs produce widespread deafferentation and reduce input to neurons across the corticothalamic system, through a dysfunction or injury of the ARAS (6–10, 29). In addition to the direct damage of the brain structures mentioned above, many authors have hypothesized a potential role of secondary mechanisms, including neuroinflammation, seizures and metabolic alteration, in influencing the onset and course of DoC (11–13). However, a reliable predictive biomarker of consciousness recovery has not been identified yet.

In this context, the effect of the thyrotropic axis on consciousness is unknown so far. Thyroid function is essential for adult brain function and yields a key role in controlling neural stem cells function in the hippocampus and the subventricular zone, main sites of neurogenesis, as well as in processes modulating neuronal plasticity after injuries (30–33). Further, THs signaling influences activation of ARAS and promotes cortical function, while TRH is known to influence the state of consciousness (19). Reports exist that consciousness can be disturbed in severe hypo- or hyperthyroidism (16–18). Yet, there are no clinical studies aiming at evaluating the potential role of thyrotropic axis in influencing the DOC secondary to ABI in patients without thyroid diseases. In this pioneering study we found that, independently from confounders, lower baseline TSH levels and higher TSH increments during 6-months inpatient neurorehabilitation predicted an improvement of DoC, while baseline fT4 and fT3 levels and their variations during rehabilitation did not appear to play a role in DoC changes. Although serum TSH levels are usually normal during the post-acute phase of ABI, we were unable to find a correlation between TSH and THs, suggesting a central derangement of pituitary TSH secretion (33). It is known that thyrotropic axis signaling in the brain can be disrupted by a severe damage (34), likely through mechanisms that involved both THs signaling deregulation and alterations in TRH and TSH secretion and feedbacks (32, 35). Like other peripheral tissues, the SNC contains deiodinases, a group of enzymes that is capable of modifying the biologic activity of THs either by activating T4 (type II deiodinase, D2) or by inactivating T4 and T3 (type III deiodinase, D3), thus modulating T3 levels inside the target cells (32). Animal studies have suggested that a severe brain insult can affect D2 and D3 expression, and modify THs signaling in localized brain areas including the cerebral cortex, which is one of the main structures involved in the complex network that regulates consciousness (34, 36–39). Although we found no associations between serum fT4 and fT3 levels and DoC evolution, we hypothesize that serum levels of THs are not able to reflect the local alterations of THs in the brain. Alternatively, an altered permeability of blood brain barrier (BBB) could allow an abnormal entry of THs from the bloodstream to the brain, but this remains difficult to be quantified (33).

To explain the relationship observed between TSH and changes in DoC, it should be reminded that the brain tissue presents a widespread expression of the TSH receptor (TSH-R), a circumstance that hypothetically implies a central function for TSH (40). A functional role for TSH-R in brain cells following brain injury is potentially supported by evidence that TSH potently and steadily stimulates D2 activity in the brain, while promoting pro-mitogenic effects in astroglia (41, 42). Thus, in the context of ABI, we speculate that the *in vivo* effect of increasing TSH during neurorehabilitation may protect the brain against prolonged neuronal stress and favour, to some extent, the recovery of injured brain cells by enhancing the conversion into active TH and by stimulating astrocyte response. The *in vivo* mechanisms mediating TSH effects on DoC remain to be clarified.

It is known that ABIs represent an important cause of death and disability (43, 44). The role of thyrotropic axis on rehabilitation and mortality after acquired brain injuries is still debated. In a previous study on patients with mild-to-severe TBI, we observed that increasing TSH and declining fT3 levels were associated with worst neurological and functional outcomes, as well as with a higher risk of mortality within 6 months following TBI (33). In this cohort, a narrower variation of fT4 levels was associated with an improvement in functional disability level and cognitive functions. We hypothesize that low variations in circulating fT4 may reflect a condition of systemic balance and limit potential excursions at the local level, rather than acting directly as a predictor of functional recovery.

Our study has some limitations which should be pointed out. First, being a retrospective study, the study is aimed to find associations, without insights into mechanisms, which require *ad hoc* investigations. Second, thyroid parameters at discharge were available for only 50 patients. Third, many methodological factors can interfere with laboratory tests used to asses thyroid function parameters. Fourth, we did not assess the evolution of the thyroid function during the rehabilitation process, and this hampers a full interpretation of the herein observed associations.

In conclusion, serum TSH levels assessed in the subacute post-ABI phase and its variation during neurorehabilitation could represent a potential biomarker of DoC evolution, while variations in fT4 levels seem to be associated with rehabilitation and cognitive functions. Further studies are needed to investigate the mechanisms underlying these associations.

## Data Availability Statement

The original contributions presented in the study are included in the article/**Supplementary Material**, further inquiries can be directed to the corresponding author.

## Ethics Statement

The studies involving human participants were reviewed and approved by Ethical Committee ICS Maugeri (#2503 CE). The patients/participants provided their written informed consent to participate in this study.

## Author Contributions

CM designed the study and draft the manuscript; AT, SB, AE, PMa and VP interpreted the results and contributed to the discussion; AT, SB, LL, DS, PMo, LM and BL collected and analyzed data; GA and AN contributed to analyse data and reviewed manuscript; PMa and VP reviewed and edited the manuscript. All authors contributed to the article and approved the submitted version.

## Funding

Paolo Marzullo is partly funded by the Italian Ministry of Health #18C201_2012.

## Conflict of Interest

The authors declare that the research was conducted in the absence of any commercial or financial relationships that could be construed as a potential conflict of interest.

## Publisher’s Note

All claims expressed in this article are solely those of the authors and do not necessarily represent those of their affiliated organizations, or those of the publisher, the editors and the reviewers. Any product that may be evaluated in this article, or claim that may be made by its manufacturer, is not guaranteed or endorsed by the publisher.
