# Supplementary material for: Thyrotropic Axis and Disorders of Consciousness in Acquired Brain Injury: A Potential Intriguing Association?
Source: Front Endocrinol (Lausanne). 2022 Jul 7;13:887701. doi: 10.3389/fendo.2022.887701 (PMC9302487; doi:10.3389/fendo.2022.887701)
Supplement: Supplementary file 1 [file Table_1.docx]

Supplementary Material

# Supplementary Table 1. Univariate linear regression analysis to evaluate the predictive role of thyroid function parameters on recovery and functional outcome.

| **Variables**  **β coefficient** | | **TSH (µIU/mL)** | | | **fT4 (ng/dL)** | | | **fT3 (pg/mL)** | | |
| --- | --- | --- | --- | --- | --- | --- | --- | --- | --- | --- |
|  |  | T0 | T1 | Δ | T0 | T1 | Δ | T0 | T1 | Δ |
| GOS-E | T1 | 0.006 | -0.181 | 0.274 | -0.110 | -0.264 | -0.258 | 0.171 | 0.266 | -0.020 |
|  | Δ | 0.076 | -0.267 | 0.236 | -0.048 | -0.008 | -0.323 | 0.128 | 0.362 | 0.082 |
| DRS | T1 | 0.071 | 0.154 | -0.252 | 0.073 | 0.210 | 0.321 | 0.010 | -0.083 | -0.074 |
|  | Δ | 0.066 | 0.197 | -0.193 | 0.064 | 0.129 | 0.322 | 0.037 | -0.002 | -0.046 |
| FIM | Tot T1 | -0.084 | -0.146 | 0.255 | -0.052 | -0.219 | -0.213 | 0.053 | 0.111 | 0.101 |
|  | Δ Tot | -0.081 | -0.140 | 0.244 | -0.053 | -0.215 | -0.224 | 0.050 | 0.115 | 0.089 |
|  | Mot T1 | -0.050 | -0.100 | 0.226 | -0.055 | -0.194 | -0.204 | 0.058 | 0.122 | 0.062 |
|  | Δ Mot | -0.054 | -0.095 | 0.225 | -0.052 | 0.187 | -0.209 | 0.056 | 0.125 | 0.058 |
|  | Cog T1 | -0.150 | -0.231 | 0.268 | -0.019 | -0.267 | -0.203 | 0.007 | 0.056 | 0.216 |
|  | Δ Cog | -0.139 | -0.251 | 0.250 | -0.030 | -0.277 | -0.240 | 0.004 | 0.061 | 0.182 |

*For abbreviation: T0, on admission; T1, at discharge; TSH, thyroid-stimulating hormone; fT4, free thyroxin; fT3, free triiodothyronine; GOS-E, Glasgow Outcome Scale-Extended; DRS, Disability Rating Scale; FIM,* *Functional Independence Measure.*
